# Diverse Effects of Women’s Employment on Fertility: Insights From Italy and Poland: Diverses conséquences de l’emploi des femmes sur la fécondité: quelques informations à partir des cas de l’Italie et de la Pologne

**DOI:** 10.1007/s10680-013-9287-4

**Published:** 2013-04-11

**Authors:** Anna Matysiak, Daniele Vignoli

**Affiliations:** 1 Institute of Statistics and Demography, Warsaw School of Economics, Ul. Madalińskiego 6/8, 02-513 Warsaw, Poland; 2 DiSIA – Dipartimento di Statistica, Informatica, Applicazioni, University of Florence, Viale Morgagni 59, 50134 Florence, Italy

**Keywords:** Women’s employment, Fertility, Role specialization, Italy, Poland, Event-history analysis, Emploi féminin, Fécondité, Spécialisation des rôles, Italie, Pologne, Analyse d’histoire de vie

## Abstract

In this paper, we look into how country-specific factors shape the interrelationship between childbearing and women’s labor supply. To this end, we compare Italy and Poland, two low-fertility countries where the country-specific obstacles to work and family reconciliation are similarly strong but which differ in the history of women’s labor supply and the extent to which couples’ material aspirations are satisfied by men’s earnings. Our findings show that women’s employment clearly conflicts with childbearing in Italy, while in Poland women tend to combine the two activities, despite the similar difficulties they face. These results challenged the standard microeconomic explanations and point to the importance of other country-specific factors, apart from conditions for work and family reconciliation, in shaping women’s employment and fertility decisions, such as economic incentives or culturally rooted behavioral patterns. Overall, our study provides thus foundations for explaining the variation in the relationship between women’s employment and fertility in an enlarged Europe.

## Résumé

Cet article étudie comment des contextes nationaux spécifiques façonnent les relations entre la procréation et l’offre de main d’œuvre féminine. Dans ce but, nous comparons l’Italie et la Pologne, deux pays ayant des faibles niveaux de fécondité, avec pour chacun d’eux d’importants obstacles liés au contexte national allant à l’encontre de la conciliation famille- travail, mais qui diffèrent quant à l’histoire de l’offre de main d’œuvre féminine et sur la façon dont les aspirations matérielles du couple sont couvertes par le salaire de l’homme. Les résultats montrent qu’en Italie l’emploi des femmes entre clairement en conflit avec la procréation, alors qu’en Pologne les femmes essayent de combiner ces deux « activités » bien qu’elles aient à faire face à des difficultés semblables. Ces résultats remettent en question les explications micro-économiques classiques et montrent que, outre les conditions de conciliation famille—travail, des facteurs propres à chaque pays, tels que des incitants économiques ou des schémas de comportements culturellement enracinés, vont jouer un rôle important dans les décisions de fécondité et de participation des femmes au marché du travail. Notre étude fournit ainsi quelques bases pour expliquer les variations de la relation entre emploi des femmes et fécondité dans l’Europe élargie.

## Introduction

The most common theoretical approach used for explaining and understanding women’s childbearing and employment choices is the microeconomic theory of fertility and women’s labor supply, as proposed by Mincer (1963) and Becker (1965). This model was traditionally built upon an assumption of a *role specialization* within a couple, which defines a situation in which a couple’s utility is maximized if a man specializes in income provision and a woman divides her time between home production and market work. The authors of the microeconomic theory acknowledged that women’s employment brings additional income, and that the income effect that arises facilitates childbearing among working women and motivates mothers to re-enter employment swiftly after the birth of a child. However, it was also underlined that women are only supplementary income providers who enter the labor market if there are no young children at home. Hence, contrary to men’s, women’s fertility and employment decisions depend additionally on opportunity costs (the price effect). The role specialization model envisioned the price effect to surpass the income effect and consequently predicted women’s employment to hinder childbearing and parenthood to jeopardize mothers’ employment.

Recently, however, the role specialization assumption of the microeconomic model has been criticized, as women have been increasingly present in the labor market all over Europe, minimizing their child-related career interruptions (e.g., Oppenheimer 1977, 1997). Women are becoming increasingly reluctant to abandon a professional career for the sake of having a family (e.g., Gutiérrez-Domènech 2004). Instead, they employ deliberate strategies aimed at combining the two spheres of life through the timing of the entry to motherhood, the spacing of children, and the sequencing of births and work episodes (Ni Bhrolchain 1986a, 1986b). Consequently, it has been argued that in modern societies, the organization of the household has been changing and women start to play an increasingly important role in contributing to the household budget (Cherlin 2000; Stevenson and Wolfers 2007; Raz-Yurovich 2012). As a consequence, the price effect of women’s employment on fertility might become less likely to surpass the income effect. An increase in the importance of women’s economic role is particularly advanced in countries where women’s work is supported by welfare policies and socially accepted (e.g., Nordic countries), as well as in countries where the material aspirations of the couple cannot be satisfied solely from men’s earnings (Cherlin 2000). The validity of the latter argument was demonstrated by Macunovich (1996) who, using US times series data, showed that the income effect of women’s wages on fertility increases with a decline in the couple’s ability to satisfy its material aspirations (e.g., during an economic slowdown).

The available micro-level research on the interrelationship between fertility and women’s employment, summarized by Matysiak and Vignoli (2008), clearly substantiates the existence of a negative impact of female employment on childbearing and vice versa, for the majority of Western industrialized economies, suggesting a predominance of the price effect over the income effect. It also demonstrates that the negative association between women’s employment and childbearing is particularly evident among mothers, likely because the opportunity costs for mothers are higher than for childless women or because the deterioration of women’s position in the labor market after childbirth makes them more vulnerable and reduces their bargaining power at home (see also Neyer et al. 2011). Most of all, however, the study by Matysiak and Vignoli (2008) reveals that the magnitude of the negative effect of women’s employment on fertility and the magnitude of the negative effect of childbearing on women’s labor market performance vary across country contexts, depending on the opportunity costs in a given country. More specifically, it was shown that women tend to postpone motherhood and avoid further childbearing, as well as to take more career breaks, in countries where mothers’ employment is less institutionally supported and less socially accepted, and where the labor market institutions have not adjusted to accommodate female labor (Adserà 2005; Del Boca et al. 2005; Gustafsson and Wetzels 2000; Mills et al. 2005; Gutiérrez-Domènech 2004; Liefbroer and Corijn 1999; Muszyńska 2007). Southern European countries have been usually pointed out as a classical example of countries where the reconciliation of family life and paid work is particularly difficult and where the conflict between women’s paid work and fertility is particularly strong (Adserà 2005; Boeri et al. 2005).

By contrast to Western industrialized countries, recent microlevel research for post-socialist countries has shown that employed women are at least as likely to give birth to the first child as the non-employed (Kreyenfeld (2004) for East Germany; Kantorová (2004) for Czech Republic; Róbert and Bukodi (2005) for Hungary; Matysiak (2009a) for Poland). These findings were obtained despite the fact that post-socialist countries experienced a strong eradication of public support for working parents after the fall of state socialism (Saxonberg and Sirovatka 2006; Szelewa and Polakowski 2008) and in many the family policy-, labor market-, and culture-related barriers to work and family reconciliation have been shown to be similarly strong as in Southern Europe (Matysiak 2011; Thévenon 2011). One explanation for this finding is a prevalence of the social norm that demands women to enter motherhood before age 30 (Perelli-Harris 2005; Potancokova 2009; Mynarska 2010). Another possibility is that women in Eastern Europe might already have been established as income providers as a result of the longer periods of women’s integration in the labor market in Eastern Europe than in Western Europe. The latter effect might be additionally reinforced by aspirations to achieve Western living standards. These aspirations may have been increasing along with EU integration, but may have been difficult to satisfy with one salary only.

The discrepancy in empirical findings between Western and Eastern Europe calls for a deeper investigation of how country-specific factors shape the interrelationship between childbearing and women’s labor supply and the role of women’s employment for childbearing. To this end, we compare two low-fertility countries, where the family-policy, labor market-related, and cultural obstacles to work and family reconciliation are similarly strong but which differ in the economic organization of the household, namely Italy and Poland. Women’s economic roles are more socially accepted in Poland than in Italy due to Poland’s history of high women’s labor force participation and lower living standards of Polish households.

Our aim is to provide a comprehensive study of the interrelationship between women’s employment and fertility. Therefore, we do not limit our investigations to any particular parity, but we trace women’s employment choices around the first and the second births, i.e., the most crucial fertility transitions in the European context. By adopting a life-course perspective, we investigate how employment affects the entry to motherhood, analyze women’s (re-)entry to employment after the first birth, and study how this transition—or the lack thereof—influences mothers’ decisions to have a second child.

Overall, in light of our premises, we expect to find a strong conflict between fertility and women’s employment in both countries, resulting from a similarly unfavorable conditions for work and family reconciliation. It should be reflected in greater postponement of the entry into motherhood among employed women than among those who do not work, long work interruptions after the first birth, and a strong negative influence of post-natal employment entry on the transition to a second child. Moreover, since the difficulties with combining paid employment and care were shown to be more pronounced for mothers than childless women, we expect to find the impact of women’s employment on second birth to be more negative than the effect of women’s work on first birth.

Nevertheless, we also anticipate clear cross-country differences in the fertility and employment trajectories women choose. Namely, we expect the negative effect of women’s employment on fertility to be weaker in Poland, both with respect to the first and second child, than in Italy where the economic role of women is adopted to a lower extent. Accordingly, we also anticipate that Polish mothers will resume paid work more quickly than Italian mothers.

The following sections present information on the Italian and Polish contexts and describe the macro-level developments in fertility and women’s labor supply in the two countries. We then present the data used, the methodological strategy employed, and the results obtained. The paper concludes with a summary and a discussion of our findings.

## Italy and Poland: The Background

### Developments in Fertility and Women’s Labor-Force Participation

Italy and Poland belong to the group of countries with the lowest fertility levels in Europe, i.e., with the period total fertility rate (TFR) below 1.4 in 2009 (Eurostat Statistics Database). The process of fertility decline started first in Italy, i.e., in the 1970s. In Poland, the first signs of a decrease appeared after the baby boom of the early 1980s. As a result of the downward trend in childbearing, the TFR plummeted to the lowest low level (below 1.3) in 1993 in Italy and in 2002 in Poland (ibid). This decline in fertility rates was partly driven by a postponement of first births, particularly in Italy, where the mean age at first birth reached 31 in 2009 compared with 26.2 in Poland (ibid). Nevertheless, quantum effects also played an important role: according to Sobotka et al. (2005), they were responsible for an around 50 % decline in the number of births over the period 1978–1996 in Italy and 1990–2002 in Poland.

Despite the fact that Italy and Poland belong to the group of countries with low fertility, they differ substantially in the past and current levels of women’s labor force participation. In Italy, the rate of women’s labor force participation is still relatively low (65 % among women aged 25–49) although it has increased markedly since the early 1970s (ILO Laborsta Database). Single-earner couples invariably create the best environment for childbearing there (Vignoli and Salvini 2008): a male partner with a stable job constitutes the “first pillar” of the household and appears crucial in determining fertility behaviors (Vignoli et al. 2011). In Poland, the women’s labor force participation rate was as high as it is currently in Italy already in the 1960s and reached nearly 80 % by the end of the 1980s (ILO Laborsta Database). After the collapse of state socialism, young women did not reduce their economic activity, despite an increase in the tensions between paid work and family. This persistence of women’s economic activity at the pre-1989 level is often interpreted as an indication of women’s strong determination to find and keep a job (Kotowska and Sztanderska 2007). Furthermore, women in Poland are also more often perceived as income providers than women in Italy: the proportion of those who believe that women and men should both contribute to household budget amounts to 25 % in Italy against 42 % in Poland (authors’ calculation on European Value Study 1999).

### Context for Fertility and Employment Choices

Attachment to Catholic values and the strong position of the Catholic Church constitute a key element that unifies Italy and Poland. According to data from the International Social Survey Programme (2008), over 90 % of citizens in both countries were raised in the Catholic religion (compared with an average of 49 % in other EU member states). In addition, 87 % of Poles and 76 % of Italians stated that they believe in God and they always have, compared with less than 60 % of respondents in 12 out of 19 EU countries. This strong attachment to Catholic values has often been seen as one of the important reasons for a delayed diffusion of new family behaviors such as cohabitation or non-marital childbearing and a profound tendency to condition childbearing on an entry to marriage (De Rose et al. 2008; Kotowska et al. 2008). In 2009, for instance, births outside marriage barely exceeded 20 % in the two countries while the EU average was 37.7 %. Only recently have the countries started to experience a slight weakening of ties with the Church, a process that is particularly visible among the younger generations, and is manifested in an increase in the incidence of marital dissolution (Salvini and Vignoli 2011) as well as in cohabitation (Matysiak 2009b). In addition, both in Italy and Poland, the incidence of childlessness has been increasing remarkably over the last years (Sobotka 2004, pp. 143–145; Matysiak and Vignoli 2010).

The strong attachment to Catholic values has not restrained the process of fertility postponement and fertility decline in Italy and Poland. The reasons for that were often found in the prevalence of the strong tensions between fertility and women’s work (Kotowska and Sztanderska 2007; Kotowska et al. 2008; Salvini 2004; De Rose et al. 2008). These tensions are pronounced in three dimensions: family policies, labor market structures, and social norms.

First, both countries are characterized by a very low supply of public childcare facilities for small children aged 0–2 (Table 1). In Poland, additionally, the supply of childcare facilities for preschoolers is far from sufficient. Instead, mothers can make use of maternity and parental leave entitlements, offered to all working mothers, irrespective of their work record. Poland is much more generous than Italy in terms of leave duration. In Italy, a 5-month maternity leave is followed by an optional parental leave of 6 months, which altogether gives 11 months. In Poland, by contrast, a mother can stay home almost 3.5 years, as she can opt for a 3-year parental leave after a 16-week maternity leave.^1^ In both countries, the parental leave is usually taken directly after the maternity leave. While the financial compensation during maternity leave is rather high (80 % in Italy and 100 % in Poland), the parental leave benefits are rather low, with the exception of women employed in the public sector in Italy (see Table 1).

**Table 1:** Contextual indicators for Italy and Poland

	Italy	Poland	EU
Childcare provision (2008)			
Children aged 0–2^**a**^ (%)	7	2	20
Children aged 3 up to school age^**a**^ (%)	93	60	80
Parental leave (2006)^b^			
Duration	6 months	36 months	–
Benefit	30 % of monthly earnings in private sector, and 80–100 % in public sector	Means-tested, flat rate at the around 15 % of the average wage in the national economy	–
Labor market structures (2006)			
Percentage who are part-time employed (aged 25–49)^**c**^	28	9	31
Percentage who cannot vary start/end of working day for family reasons (aged 25–49)^d^	25	42	27
Women’s unemployment rate relative to men’s (persons aged 25–49)^**c**^	1.7	1.2	1.3
Social norms			
Percentage who agree with the statement that a working mother cannot establish just as warm and secure a relationship with her children as a mother who does not work (computation for 1999)^e^	46	36	24
Percentage who agree with the statement that a woman should work full-time (1) if children are of preschool age, (2) after the children leave home (computation for 1994)^f^	(1) 5	(1) 11	(1) 9*
	(2) 55	(2) 80	(2) 73*

*The average indicator does not refer to EU countries, but was computed for all countries that participated in ISSP 1994 survey: Australia, Austria, Bulgaria, Canada, Czech Republic, Germany, Great Britain, Hungary, Ireland, Israel, Italy, Japan, the Netherlands, New Zealand, Northern Ireland, Norway, Poland, Russia, Slovenia, Spain, Sweden, and the United States

^a^Saraceno and Keck (2008)

^b^Moss and Walls (2007)

^c^Eurostat Statistics Database (Labor Force Survey data)

^d^Eurostat Statistics Database (data from the “Reconciliation between work and family life” survey 2005)

^e^Computation on European Values Study (1999)

^f^Treas and Widmer (2000) on ISSP 1994

Second, in both countries, the labor market structures create certain barriers to women’s employment due to rigid working hours, scarcity of part-time jobs, and a strong insider–outsider divide. Poland stands out in Europe for having particularly rigid work arrangements in terms of low part-time employment and inflexibility of working hours (Table 1). A typical feature of Italy is strong barriers to women’s employment, which is reflected in large gender gaps in unemployment (see also Adserà 2005).

Finally, in both countries, the gender division of tasks is heavily asymmetric, and the social disapproval of mothers who work when their children are young is widespread (e.g., Mencarini and Tanturri 2006; Muszyńska 2007; Philipov 2008; Lück and Hoffäcker 2003; see also Table 1). This traditional perception of women as major care providers is a paradox of Poland where women are expected to withdraw from the labor force when their children are young, but to resume employment after their children get older in order to contribute to the household budget (Lück and Hoffäcker 2003; Treas and Widmer 2000).

All in all, while Italy and Poland share numerous similarities in the family policy- and labor market-related context of fertility and women’s employment, they differ widely in their levels of economic development. Despite a clear and continuous improvement in the economic situation since the early 1990s, the financial situation of Polish households is still worse than in Italy: the annual total disposable income in purchasing power standards (PPS) of a family with two working spouses (each earning the average national salary) and two children is twice as low in Poland as in Italy and the consumption expenditures of families with dependent children are three times lower (Eurostat Statistics Database 2010 on the basis of the EU-SILC data). This gap in family material situation likely means that material aspirations of the Poles are unsatisfied to a larger extent than those of Italians. In fact, only 9.1 % of Polish respondents to the European Social Survey (2002) reported that they live comfortably on their present income, compared with 34.2 % of Italians (authors’ calculations). The lower economic standing of Polish families and larger dissatisfaction with living standards might constitute some of the reasons for the strong determination of Polish women to participate in the labor force.

## Empirical Strategy

### Fertility and Women’s Employment as Parallel Careers

Fertility and women’s employment are dynamic careers that develop continuously over an individual’s life-course. Therefore, a life-course perspective is best-suited to analyzing the interrelationship between them (see also Willekens 1991). This approach allows us to trace women’s employment choices around the most crucial fertility transitions, namely the first and second births.

It has been demonstrated in the literature that decisions about fertility and women’s employment are made simultaneously (Ermisch 2003; Engelhardt et al. 2004; Del Boca et al. 2005). This implies that the two careers are jointly determined by a set of exogenous factors. A failure to account for important fertility and women’s employment determinants leads thus to a selection bias to the estimated effect of women’s employment on fertility. This bias might be upwards or downwards. The former is observed in case of a positive selection, i.e., when the unmeasured characteristics affect employment and fertility decisions positively, for instance for unobserved reasons a woman decides to start a job and have a child afterwards. In such a situation, a failure to account for these unmeasured reasons could lead us to the wrong conclusion that women’s employment facilitates childbearing. Conversely, if employment and fertility are influenced by unobserved factors in opposite directions, the effect of employment on fertility will be downwardly biased. For instance, if a woman decides to exit employment and to have a child while non-employed for reasons unknown to the researcher, a failure to account for these unmeasured reasons will lead to a wrong conclusion about the negative impact of employment on fertility.

In this study, we are interested in analyzing the effects of women’s employment on fertility instead of providing a description of fertility patterns by employment status. This means we cannot rely on the coefficient of women’s employment on fertility estimated only net of the observed characteristics of a woman, but should also account for a set of unobserved characteristics. Controlling for time-variant characteristics usually requires the use of instrumental variables, i.e., variables that are exogenous to woman’s employment but highly correlated with fertility. Applying this methodology to this research problem has been demonstrated in the literature to be highly complicated: instrumental variables are either not available or their use results in a serious sample selection (for a review see Del Boca and Locatelli 2006). Given these analytical problems in our study, we decided to limit ourselves to accounting only for time-invariant unobserved characteristics, such as woman’s specific time-constant orientation towards work or family. To this end, we estimated women’s paid work and fertility jointly in a common maximum likelihood framework. Building upon Lillard et al. (1995) and Lillard and Panis (1996), this approach has been already successfully used in this field of research (Aassve et al. 2006; Matysiak 2009a). Even though it needs to be born in mind that our estimates may be still biased by selection effects due to time-varying unmeasured factors, controlling for time-constant unobserved characteristics of women is already a substantial improvement over the conventional event-history applications.

### Data

In our empirical investigation, we employed retrospective data stemming from the Household Multipurpose Survey Family and Social Subjects (FSS), which corresponds to the Italian Generations and Gender Survey, and the Polish Employment, Family and Education Survey (EFES). The Italian survey was conducted by the Italian National Statistical Office (Istat) in November 2003 on a sample of about 24,000 households and 49,451 individuals of all ages. The Polish survey was prepared by the Institute of Statistics and Demography of the Warsaw School of Economics and carried out in November and December 2006 on a representative sample of 3,000 women born from 1966 to 1981. These two retrospective surveys represent comprehensive sources of data available for the two countries. While both surveys cover detailed information on women’s fertility and employment histories recorded on a monthly basis, they also have several limitations that restrict our analytical options. First, neither of these surveys contains data on income from work. Hence, even though such data would be very useful for investigating the income effect of women’s wages on fertility, we need to limit ourselves to investigating the interrelationship between women’s employment and childbearing. Second, there are also some differences between the two studies that restrict the possibility of fully exploiting data potentials in a comparative project. In contrast to the Italian survey, the EFES focused on women, and was conducted on selected cohorts only. This prohibits us from carrying out analyses at a couple level, and limits opportunities for studying temporal changes in individual behavior. Conversely, the FSS, unlike the EFES, does not contain information on unemployment spells and changes in work contracts throughout respondents’ employment histories, which forces us to analyze the interrelationship between childbearing and women’s employment status in general (employed versus not employed). Finally, unlike the Polish EFES, the Italian FSS does not provide information about whether a woman is on parental leave. More specifically, women taking part in the survey could classify themselves either as employed or as non-employed. Given that mothers in Italy can be out of paid employment no longer than 11 months following birth without risking the loss of their work contract (a 5-month maternity leave is followed by an optional parental leave of 6 months), we lack reliable information on women’s employment status within the first year after birth in Italy. We deal with this problem by coding the episode of first year after birth as missing in women’s employment histories in both countries. The remaining episodes of parental leave in Poland are coded as non-employment (for an explanation see Matysiak 2009a). Finally, the datasets we use do not allow us to introduce some important control variables into our models, for example the religiosity, which might be crucial in the Italian and Polish contexts. In the EFES, this variable is not available at all, and in the FSS it was collected at the time of the interview only, which precludes its use in the dynamic model without encountering problems of ‘anticipatory analysis’ (e.g., Hoem and Kreyenfeld 2006).

Given our data limitations, we focused on analyzing the interrelationship between women’s employment status in general (employed vs. not employed) and fertility. For Poland, we selected the female cohorts born between 1970 and 1981. These women were between the ages of 8 and 19 in 1989, which means that they started their reproductive careers largely under the new political and economic conditions. Taking the same cohorts for Italy as for Poland would mean following the Italian women for a period that is 3 years shorter than the period for Poland. For this reason, for Italy we chose cohorts born in the years 1967–1978. As a result, in both cases the analyzed women were aged 25–36 at the time of the interview. Importantly, we did not censor Polish women in 2003 (i.e., the date of Italian interview). The rationale behind this choice was that because fertility postponement in Poland started only recently, the years 2004–2006 could provide us with valuable information.

From the original sample we excluded women who reported twins at the first birth. Respondents with missing values on other variables (i.e., parents’ education) were retained in the sample, and additional modalities “missing” were created for these covariates. As a result, our Italian final sample included 4,238 respondents, and the Polish final sample covered 2,300 respondents.

## Methods

*Effects of women’s employment on fertility.* Two hazard models for the transition to the first and second birth were specified in the first step in order to study employment effects on fertility. Each woman was followed from the age of 15 until the first conception, and then from the delivery of the first child up to the conception of the second child; cases were eventually censored at the date of the interview. Conception was measured 7 months before birth, i.e., at a point at which the great majority of women are aware that they are pregnant, and after which this knowledge may influence their subsequent employment behaviors.

The choice to observe a woman since the age of 15 instead of following her since union formation, when she in fact becomes at risk of falling pregnant, was made deliberately as we believe it to be more reasonable in the context of Italy and Poland. Childbearing and marriage are tightly bound in the two countries. It is very common for the conceptions to take place within the first 6 months after partners move in together (mainly form a marriage) as they likely do so with a plan to have a child. In our datasets, we only have information on the dates of forming a cohabitation/marriage, but no information on partnership episodes preceding co-residence. We thus believe that in such circumstances estimating a duration model with a process time beginning at the start of the co-residence is a wrong strategy because the process of deciding about the child often begins earlier.

The hazard models for the first and second birth were estimated jointly, with a common unobserved heterogeneity term, which allowed us to account for selection of family-oriented women to the sample used for studying second births. Since we were interested in investigating the effects of employment on birth transitions, employment status was our main explanatory covariate. In order to control for the time-constant unobserved characteristics of women, in the second step we modeled fertility transitions jointly with employment transitions allowing for a correlation between the unobserved person-specific characteristics affecting fertility and employment behaviors (e.g., Lillard et al. 1995; Lillard and Panis 1996). Consequently, we needed to specify a model for employment transitions around the first birth.

Modeling employment transitions around the first birth was complex due to the data limitations we encountered; namely, the lack of information on women’s employment status within the first year after the first birth in Italy. Given this limitation, we decided to specify three employment equations, which were modeled simultaneously with a common unobserved heterogeneity term: (1) a logit model for the probability of being employed at first conception, (2) a logit model for the probability of being employed 12 months after first birth and (3) a hazard model for entry to work with a baseline duration, represented by the time elapsed since the child was 1 year old until a woman entered work. The first equation (i.e., the logit model for the probability of being employed at conception) was estimated to control for selection of women with a work contract to the sample of women who entered paid work relatively quickly after the first birth (at least in Italy, these were women who had the right to parental leave) and selection of women with no work contract to the sample of women who remained out of work after that time. The second equation (i.e., the logit model for the probability of being employed 1 year after first birth) was specified in order to account for selection of family-oriented women to the sample of women who were still at home 12 months after birth.

In order to analyze the effects of employment on fertility transitions, we estimated simultaneously the following five equations:

$$ \left\{ {\begin{array}{*{20}c} {\ln h^{B1} (t) = y^{B1} (t) + \sum\nolimits_{j} {a_{j}^{B1} X_{j} + \sum\nolimits_{i} {b_{i}^{B1} Z_{i} (t) + \varepsilon } } } \hfill & {\text{ Hazard of first conception}} \hfill \\ {\ln h^{B2} (t) = y^{B2} (t) + \sum\nolimits_{j} {a_{j}^{B2} X_{j} + \sum\nolimits_{i} {b_{i}^{B2} Z_{i} (t) + \varepsilon } } } \hfill & {\text{ Hazard of second conception}} \hfill \\ {\ln (\hat{Z}) = a_{{_{0} }}^{C} + \sum\nolimits_{j} {a_{j}^{C} X_{j} + \eta } } \hfill & {\text{ Logit for work at conception}} \hfill \\ {\ln (\hat{V}) = a_{{_{0} }}^{B} + \sum\nolimits_{j} {a_{{_{j} }}^{B} X_{j} + \eta } } \hfill & {\text{ Logit for work 12 months after birth}} \hfill \\ {\ln h^{EN12 + } (t) = y^{EN12 + } (t) + \sum\nolimits_{j} {a_{j}^{EN12 + } X_{j} + \sum\nolimits_{i} {b_{i}^{EN12 + } Z_{i} (t) + \eta } } } \hfill & {\text{ Hazard of employment entry}} \hfill \\ \end{array} } \right. $$

For simplicity, the subscripts standing for an individual were omitted. The ln(hB1), ln(hB2), and ln(hEN12 +) denote natural logarithms of hazards of first birth, second birth and entry to employment starting 12 months after the first delivery, respectively. $\hat{Z}$ represents the odds of working at conception and $\hat{V}$ the odds of entering employment when the child is 1 year old. In each of the hazard models, $y(t)$ stands for a piecewise linear spline that captures the effect of the baseline duration on analyzed intensities; i.e., age of a woman in the model for the transition to the first birth, and age of the first child in the model for the transition to the second child and employment entry.^2^ The vector $X_{j}$ denotes time-constant covariates, and the vector $Z_{j} (t)$ the time-varying covariates. Finally, $\varepsilon$ and $\eta$ stand for person-specific unobserved heterogeneity terms, which are constant across women’s fertility and employment spells, respectively. Consequently, $\varepsilon$ captures the unobserved time-invariant propensity of women to have a child, while $\eta$ accounts for women’s unmeasured time-constant determination to participate in paid employment. Both unobserved heterogeneity terms were assumed to follow normal distribution with zero means and standard deviations of $\sigma_{\varepsilon }^{{}}$ and $\sigma_{\eta }^{{}}$:

$$ \left( {\begin{array}{*{20}c} \varepsilon \\ \eta \\ \end{array} } \right) \sim N\left( {\left( {\begin{array}{*{20}c} 0 \\ 0 \\ \end{array} } \right),\left( {\begin{array}{*{20}c} {\sigma_{\varepsilon }^{2} } & {\rho_{\varepsilon \eta } } \\ {\rho_{\eta \varepsilon } } & {\sigma^{2}_{\eta } } \\ \end{array} } \right)} \right) $$

where $\rho_{\eta \varepsilon }$ $\left( { \equiv \rho_{\varepsilon \eta } } \right)$ represents the correlation between the unobserved heterogeneity terms. This correlation is significant if the two processes are affected simultaneously by unobserved factors. It is positive in case of a positive selection, and negative otherwise. The identification of the model is attained through within-person replication—in this specific context, many women have had more than one birth as well as several employment episodes (Lillard et al. 1995, p. 446).

Our key explanatory covariate in the fertility models is woman’s employment status, introduced as a time-varying covariate. In the model for the transition to first child, this variable is grouped into four categories: “in education”, “in first non-employment, but out of education”, “employed” and “in higher order non-employment”. This specification allows us to separate women who are still in school from those who have already graduated and face the decision of whether or not to enter the labor market. It also separates women who ever worked from those who are in their first non-employment spell. In the model for the transition to the second child, the employment status variable has the following categories: “entered no work after first birth” (including women on parental leave in Poland), “entered work after first birth, but currently in non-employment”, and “entered work after first birth and currently in employment”. Again, we separated non-employed women who had not entered employment after delivery from those who had taken a paid job, but either lost it after some time or decided to give it up.

Additionally, all models control for the respondent’s and the parents’ education, as well as for calendar time. Respondent’s education is a time-varying covariate. Women who had finished their education were classified into three groups: low, medium, and high. The first category comprises women who completed only compulsory education (8 years in both countries), as well as those who continued with basic vocational education, lasting 3 years in Italy and 2 years in Poland. The medium educated are those who completed at least 4 years of education at the upper-secondary level, as well as those who undertook post-secondary but non-tertiary education. Women who received a bachelor’s or a master’s degree were classified as highly educated. Parent’s education were dichotomised between “low” and “medium–high”. Finally, the logit models predicting the probability of being employed at conception and 1 year after birth, as well as the hazard model for employment entry, account for work experience gathered before the first conception, as well as the number of non-employment spells experienced until that moment.

We deliberately abstain from including woman’s partnership status as a control variable in our analyses. Union formation and fertility are jointly determined. As our initial analyses (not presented here) demonstrated this is particularly true in the Italian and Polish contexts where cohabitation is rare and individuals usually marry no sooner than when they plan to become parents. This implies that considering partnership status just as covariate in the fertility equation largely captures the influence that some other covariates have on fertility, biasing the estimates of interest. Partnership status could be accounted for only through a joint estimation with fertility process (see also Aassve et al. 2006), which is far from the scope of this paper.

*Entry to employment after first birth.* In order to investigate the patterns of women’s employment entry after first birth, we compared Kaplan-Maier survival curves for the two countries. Normally, in such a situation it would make the most sense to observe a woman since her first birth. This approach was not appropriate in our case, however, as we lacked reliable information on women’s employment status within the year after birth in Italy. Hence, we could not know the date when Italian mothers finished parental leave and re-entered paid work. For this reason, we decided to observe each woman starting with the first birthday of the first child. Women were followed until the date of employment (re-)entry, with censoring at the second conception or the time of the interview, whichever came first. The resulting Kaplan–Meier estimates of survival curves were multiplied by the proportions of women at work 1 year after the first birth in order they could reflect proportions of women back to paid work at any moment t after the birth of the child.

## Empirical Results

We first present our estimates of employment effects on the transition to first birth. Subsequently, we show descriptive information on the patterns of women’s entry to employment after the first delivery in order to illustrate the employment situation of mothers prior to the possible decision to have the second child. Finally, we turn our attention to the impact of women’s employment on second birth risks. Each time we discuss the estimates of employment effects on fertility, we refer first to the descriptive findings that come from the simple intensity models for first and second births (modeled separately from employment transitions). These simple hazard models for first and second births are presented in columns (2) in Tables 2–3 and provide information about fertility differentials with respect to women’s employment status. Only then do we move on to interpreting the employment parameters from the joint models, i.e., net of selection effects, which are stored in columns (3) in Tables 2–3. The full model results are reported in the Appendix.

**Table 2:** Impact of women’s employment status on the transition to first conception

Employment status	Single process model	Joint model
(1)	(2)	(3)
Italy		
Student	0.27***	0.29***
First non-employment	1	1
Second or higher non-employment	1.05	1.08
Employment	0.69***	0.62***
Poland		
Student	0.68***	0.69**
First non-employment	1	1
Second or higher non-employment	1.1	1.11
Employment	1.06	1.02

* 10 %; ** 5 %; *** 1 %

Results are controlled for woman’s age, educational level, calendar period, and woman’s social background (parents’ educational level)

*Source:* own elaboration on FSS (2003) and EFES (2006)

**Table 3:** Impact of women’s employment status on the transition to the second conception

Employment status	Single process model		Joint model	
(1)	(2)		(3)	
Italy				
Did not enter work after first birth	1		1	
Entered work and is currently working	0.83**		0.67***	
Entered work but is not currently working	1.2		1.07	
Poland				
Did not enter work after first birth	1		1	
Entered work and is currently working	1.07		0.85	
Entered work but is not currently working	0.79		0.62**	

* 10 %; ** 5 %; *** 1 %

Results are controlled for age of the first child, woman’s age at first birth, woman’s educational level, calendar period, and woman’s social background (parents’ education)

*Source:* own elaboration on FSS (2003) and EFES (2006)

### Impact of employment on the first birth

Our descriptive outcomes, presented in column (2) in Table 2, point to clear differences in the employment and motherhood choices of Italian and Polish women. In the former country, employed women were found to be far less likely to conceive a first child than women who had never worked or women who had worked in the past, but who had since exited the labor market. For Poland, we found no relationship between women’s employment and the transition to a first birth. Specifically, Polish women who had a job were shown to be as likely to enter motherhood as those out of paid work.

The correlation between the unobserved heterogeneity terms in fertility and employment equations was significant and positive. This suggests that unobserved time-invariant factors influence childbearing and employment entry in the same direction. The simultaneous modeling of fertility and women’s employment appear thus to be strongly justified because after we controlled for the time-invariant unobservables, the estimated coefficients of the effect of employment on first birth became lower (see column (3) in Table 2). After accounting for time-invariant unobserved characteristics, the negative effect of employment on the first birth risk in Italy became slightly stronger. The effect in Poland remained insignificant. Overall, our findings suggest that employment clearly discourages motherhood in Italy, but has no effect on first births in Poland.

### Timing of employment (re-)entry after first birth

**Fig. 1:**
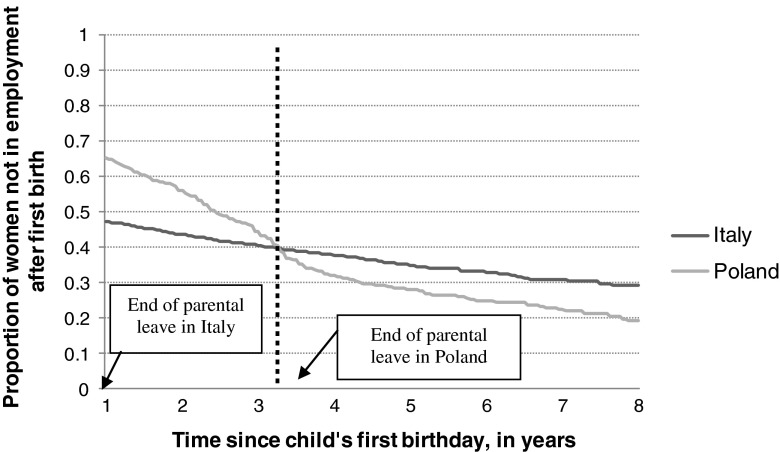
Proportion of women not in employment 12 months after the first birth. Kaplan–Meier survival curve estimates. *Source:* own elaboration on FSS (2003) and EFES (2006)

In this sub-section, we describe the timing of employment (re-)entry after the first birth through Kaplan–Meier survival curve estimates. These curves were corrected by the proportions of women at work 1 year after the first birth. Although, unlike in Poland, employment strongly discourages the transition to motherhood in Italy, Italian mothers resume employment after their first birth more quickly than Polish women (Fig. 1). Half of Italian mothers, but only one-third of Polish mothers, were working 1 year after the birth. This difference in entry rates can be due to differences in parental leave regulations. In fact, it turns out that the intensity of employment entry in Poland starts to exceed that of Italy after the child reaches 3.5 years of age, which is precisely the time when the parental leave expires.

**Fig. 2:**
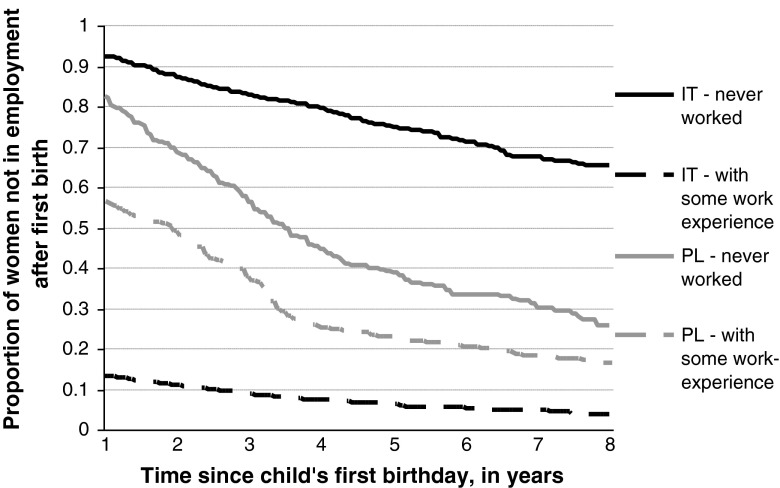
Proportion of women not in employment 12 months after child’s first birthday, by women’s work experience before the first birth. Kaplan–Meier survival curve estimates. *Source:* own elaboration on FSS (2003) and EFES (2006)

It turns out, however, that the intensity of employment entry in Italy strongly depends on whether a woman was employed for at least some period of time before the first conception (Fig. 2). Almost 90 % of the Italian women who had some work experience (who made up 58 % of our sample) entered employment 1 year after giving birth. By contrast, of those women who had never worked before they conceived, no more than 10 % were employed 1 year after the first birth, and only 20 % were in a job 3 years later. These findings indicate there is a strong polarization in the behavioral patterns of Italian women. This degree of strong polarization was not observed in Poland, although women with some work experience (who constituted almost 70 % of all the Polish women in our sample) were found to be more likely to enter paid work after childbirth than those who had never worked.

### Impact of employment entry on second birth

In the last step, we investigated how employment transitions after the first birth influence the progression to the second child. Our descriptive findings from the single-process models, illustrate clear cross-country differences (see column (2) in Table 3). In Italy, women who entered work after the first birth and remained employed were 19 % less likely to conceive a second child. The direct effect—i.e., after controlling for selection—was even stronger (35 %, see column (3) in Table 3), suggesting that employment after first birth in Italy clearly hinders the transition to second child. Interestingly, the negative effect of women’s employment on second birth does not seem to be more pronounced than the effect of employment on first birth. By contrast to currently working women, those women who took up a job but left it after some time for unknown reasons were as likely to have conceived a second child as women who had not entered employment following the first birth.

In Poland, the picture is completely different. Both the simple model, without accounting for selection effects, as well as the multi-process hazard model suggest that women who entered employment were as likely to have a second child as women who had not done so. It is to be noted that a similar, insignificant, effect of women’s employment on childbearing was also reported among childless women. Furthermore, conversely to Italy, women who entered employment after they gave birth to the first child, but left the workforce for unknown reasons, were less likely to conceive a second child than those who had not entered employment after birth (this effect became significant after accounting for the endogeneity between fertility and employment). More specifically, exiting the labor market after some episode of employment after birth reduces the risk of second conception by 38 %. One possible explanation for this finding is that women who entered employment after the first birth, but exited it after a period of time, were terminated by their employer. If so, our results for Poland suggest that, employment entry after the first birth does not significantly conflict with further childbearing, but the involuntary loss of a job creates particularly unfavorable conditions for enlarging the family size.

### Sensitivity analyses

Our data had some important shortcomings, namely we missed the information on the use of parental leave for Italy and we were not able to differentiate between the status of an unemployed and a housewife among the Italian women. As this information was present in the Polish dataset we tested how its use would affect our findings.^3^ First, we exploited the information on women’s employment status within the first year after birth by replacing the probit model for the probability of employment 1 year after birth and the hazard of employment entry after child’s first birthday in our joint model by a hazard model of employment entry after first birth. The results remained virtually unchanged which lends credibility to our research design of dealing with the missing information on the parental leave use in Italy. Second, we differentiated between the status of a housewife, an unemployed and being on a parental leave in the models for the transitions to first and second birth. Distinguishing the effect of unemployment from the effect of inactivity was particularly important in the Polish context due to the high unemployment pressure in the country in the analyzed time period. The model suggests that the transition to first conception in Poland is not affected either by employment or unemployment while the risk of second conception is significantly reduced by the fact of being unemployed. Furthermore, no significant differences in the risk of second conception between inactive women, women on parental leave and those who entered employment after first birth were revealed. We found these results to be largely consistent with those presented in the previous sections of the paper: contrary to an involuntary job loss, paid employment does not constitute a barrier to childbearing in Poland.

### Summary of empirical findings

Overall, our empirical findings point to clear cross-country differences in the effects of women’s employment on childbearing. Namely, they suggest that women’s employment constitutes a clear barrier to childbearing in Italy, while it does not conflict with fertility in Poland.

In Italy, employed women are very likely to postpone the entry to motherhood. They also are highly unlikely to give birth to a second child, the magnitude of which effect is similarly large as the magnitude of women’s employment on first birth. Instead of enlarging the family size, Italian mothers swiftly return to employment after the first birth. This pattern of rapid employment re-entry in Italy is likely driven by the rather modest parental leave provisions (at least in terms of parental leave duration). Overall, the life course trajectory described above was taken by around one-half of the Italian women born 1967–1978, and the choice to take this trajectory seems to have been determined to a large extent by whether or not a woman decided to pursue a career after she graduated. The remaining women, who did not enter employment after completing education, followed a completely different life path: i.e., they tended to progress to having a first child more quickly than those who worked, they seldom took a job after giving birth and they had the highest intensity of progressing to a second child. A polarization in women’s fertility and employment behaviors is hence a clear feature of the Italian setting (see also Saurel-Cubizolles et al. 1999; Gutiérrez-Domènech 2004 for the older Italian cohorts).

In Poland, where the family- and labor market-related as well as cultural setting seems to be at least as unfavorable to work and family reconciliation as in Italy, women’s employment did not seem to constitute a barrier to fertility. More specifically, women’s paid work was not found to significantly affect either the entry to motherhood nor the progression to a second child. Moreover, in Poland, a job loss after re-entry was shown to have led to delays in the second birth, which contrasts with the finding for Italy. Polish women stay home longer than Italian women after the first birth, very likely because of a longer parental leave entitlement. The majority return to paid employment, however. Unlike in Italy, no polarization pattern in fertility and employment behaviors was observed. Altogether, women’s employment seems to conflict with childbearing in Italy, while in Poland women tend to combine the two activities, despite the difficulties they face.

## Discussion

Women’s fertility and employment choices have been widely studied in demographic, economic, and sociological literature. In general, empirical studies for Western industrialized countries suggest that the two careers are in conflict, but that this conflict is weaker when there is institutional support for employed parents, when the labor market is adjusted to accommodate female labor and when the social acceptance of working mothers is high. These findings are consistent with the microeconomic theory of fertility and women’s labor supply that presupposes women’s fertility and employment choices to be largely determined by opportunity costs. Recent micro-level research on post-socialist countries has challenged this prediction, however, by showing that working women in this part of Europe are not less likely to enter motherhood than those who do not have a job, even if the country context is not supportive of work and family reconciliation. This may suggest that conditions for work and family reconciliation are not the only country-specific factor affecting women’s fertility and employment behaviors; and that women’s employment may be an important facilitator of family formation in countries with longer histories of women’s labor force participation, where women are accepted as income providers.

In this paper, we looked more closely into how country-specific factors shape the interrelationship between childbearing and women’s labor supply by comparing Italy and Poland, two low-fertility countries where the country-specific obstacles to work and family reconciliation are similarly strong but which differ in the history of women’s labor supply and the extent to which couple’s material aspirations are satisfied by men’s earnings. Comparing these two countries provided us thus with an opportunity for studying how the differences in women’s economic roles that permeated the past affect contemporary women’s employment and fertility behaviors. Our findings indeed showed that women’s employment clearly conflicts with childbearing in Italy, while in Poland women tend to combine the two activities, despite the difficulties they face. Overall, our study provides thus new insights into the interrelationship between fertility and women’s employment in an enlarged Europe.

Our paper also has an important methodological contribution as it demonstrates that one should account for the unobserved person-specific characteristics which jointly affect women’s fertility and women’s employment behaviors. In this study we succeeded in considering the time-constant characteristics which were not present in our data, such as for instance work- or family-orientation or religiosity. By estimating fertility and women’s employment jointly in a common maximum likelihood framework we showed that a failure to account for the unmeasured time-constant unobserved characteristics of women would lead to an underestimation of the negative impact of women’s employment on first and second birth risks.

This study does also have some clear data-related caveats and requires some extensions. Most of all, even though we accounted for the time-constant unobserved characteristics of women we were not able to control for the time-varying unobserved factors that might affect women’s fertility and employment choices, such as changes in individual values or preferences. Furthermore, as we could not introduce the economic situation of the household and partners’ earnings or the labor market situation of the male partner, we could not investigate how they affect women’s childbearing behaviors. Consequently, the effects of women’s paid work on fertility we found, although closer to the real ones than those obtained from simple single-process hazard models, are not necessary causal.

Despite these limitations, our findings clearly challenged the standard microeconomic explanations and point to the importance of other country-specific factors, apart from conditions for work and family reconciliation, in shaping women’s employment and fertility decisions, such as economic incentives or culturally rooted behavioral patterns, such as social norms on mothers’ involvement in paid work or social norms on the optimal age at first birth. These factors might be particularly important for explaining the fertility—employment puzzle in Eastern Europe. Moreover, the economic incentives of women’s paid work might gain on importance also in Western Europe given the continuous increase in women’s labor force participation as well as the rising instability of men’s income, particularly at the times of the worldwide economic recession. Future comparative research, involving larger number of countries and using longitudinal data on fertility and couples’ earnings, should therefore look more closely at the role of economic factors on partners’ fertility behaviors in order to test this claim as well as investigate the effects it might have on fertility across time and space.

## Appendix

See Table 4.

**Table 4:** Full model results: coefficients, standard errors, and significance levels

		ITALY						POLAND					
		Single process model			Multiprocess model			Single process model			Multiprocess model		
		Coeff.	Sig.	St. Er.	Coeff.	Sig.	St. Er.	Coeff.	Sig.	St. Er.	Coeff.	Sig.	St. Er.
*Transition to first conception* Age (spline), ref = 15													
Constant		−6.79	***	0.36	−7.63	***	0.52	−5.55	***	0.24	−5.97	***	0.34
15–20		0.40	***	0.05	0.43	***	0.06	0.46	***	0.04	0.49	***	0.05
20–24		0.18	***	0.03	0.22	***	0.04	0.12	***	0.03	0.18	***	0.04
24–28		0.16	***	0.03	0.20	***	0.03	0.02		0.04	0.08	*	0.04
28–32		0.03		0.03	0.07	*	0.04	−0.04		0.06	0.00		0.08
32–37		−0.16	**	0.07	−0.13	*	0.08	−0.38	*	0.21	−0.36		0.23
Calendar time (spline), ref = 2003													
Spline		−0.04	***	0.01	−0.05	***	0.01	−0.04	***	0.01	−0.04	***	0.01
Educational attainment (splines)													
Tertiary													
Exiting education (shift)		−1.11	***	0.39	−1.10	**	0.47	0.14		0.18	0.11		0.19
0–4 years (slope)		0.44	***	0.10	0.45	***	0.10	0.14	**	0.07	0.15	**	0.07
4+ years (slope)		0.09	*	0.05	0.14	**	0.06	0.01		0.09	0.02		0.10
Secondary													
Exiting education (shift)		−0.53		0.33	−0.39		0.41	0.84	***	0.14	0.87	***	0.16
0–4 years (slope)		0.27	***	0.07	0.26	***	0.07	−0.06		0.04	−0.05		0.05
4+ years (slope)		0.05	**	0.02	0.09	***	0.03	−0.03		0.04	−0.02		0.04
Primary													
Exiting education (shift)		0.56		0.45	0.77		0.53	1.21	***	0.19	1.19	***	0.22
0–2 years (slope)		0.39	**	0.19	0.36	*	0.21	−0.03		0.09	0.05		0.10
2+ years (slope)		−0.03	*	0.02	0.01		0.02	−0.11	***	0.02	−0.10	***	0.03
Employment status (ref = first non employment)													
Still in education		−1.32	***	0.27	−1.25	***	0.40	−0.39	***	0.14	−0.37	**	0.19
Second or higher non-employment		0.05		0.07	0.08		0.09	0.09		0.09	0.10		0.12
Employment		−0.37	***	0.05	−0.48	***	0.08	0.06		0.06	0.02		0.09
Father’s education (ref = medium–high)													
Low		0.18	**	0.09	0.20	*	0.12	0.19	**	0.08	0.22	**	0.09
Mother’s education (ref = medium–high)													
Low		0.03		0.10	0.07		0.13	0.13	*	0.07	0.19	**	0.09
*Transition to second conception*													
Time elapsed since first birth (spline), ref = 0													
Constant		−5.25	***	0.47	−5.93	***	0.55	-4.71	***	0.36	−4.91	***	0.49
0–1		3.28	***	0.42	3.35	***	0.46	2.45	***	0.32	2.53	***	0.43
41334.00		0.21	***	0.07	0.34	***	0.07	0.10		0.07	0.20	***	0.08
3+		−0.05		0.04	0.07		0.05	−0.12	**	0.05	−0.05		0.05
Calendar time (spline), ref = 2003													
Spline		−0.02		0.01	−0.02		0.02	−0.05	***	0.01	−0.06	***	0.01
Age at previous birth (ref = 20–22)													
15–20		0.09		0.13	−0.32		0.24	0.17		0.12	−0.20		0.22
20–26		−0.02		0.10	−0.26	*	0.15	0.21	**	0.10	0.00		0.14
26–30		0.04		0.09	0.33	**	0.15	−0.12		0.14	0.09		0.18
30+		−0.20		0.15	0.43	*	0.25	−0.74	*	0.42	−0.31		0.46
Educational attainment (ref = primary)													
Tertiary		0.44	***	0.17	0.05		0.27	−0.37	**	0.15	−0.58	***	0.20
Secondary		0.12		0.08	−0.12		0.13	−0.21	**	0.08	−0.31	***	0.11
Still in education		−0.33		0.25	−0.80	**	0.33	−0.71	***	0.18	−0.87	***	0.21
Employment status (ref = did not enter work after first birth)													
Entered work and is currently working		−0.19	**	0.08	−0.39	***	0.11	0.07		0.08	−0.16		0.12
Entered work but is not currently working		0.18		0.25	0.06		0.30	−0.24		0.20	-0.48	**	0.23
Father’s education (ref = medium–high)													
Low		0.17		0.15	0.30		0.19	0.11		0.11	0.16		0.14
Mother’s education (ref = medium–high)													
Low		−0.21		0.16	−0.35	*	0.20	0.17		0.11	0.23	*	0.13
*Work at conception*													
Constant					−4.21	***	0.59				−1.17	***	0.30
Calendar time (ref = 1998–2003)													
<1994					0.12		0.34				−0.11		0.22
1994–1998					−0.06		0.35				0.00		0.18
2003–2006					–	–	–				−0.34		0.26
Educational attainment (ref = primary)													
Tertiary					2.86	***	0.66				1.92	***	0.37
Secondary					0.86	***	0.30				0.59	***	0.19
Still in education					2.88	***	0.64				-0.14		0.28
Cumulated work experience (ref = never worked)													
(0–2) years					5.66	***	0.52				2.78	***	0.26
(2–4) years					7.00	***	0.57				3.35	***	0.28
(4–6) years					8.34	***	0.72				3.64	***	0.36
(6–8) years					8.18	***	0.67				4.87	***	0.66
>8 years					9.20	***	0.73				4.15	***	0.60
Father’s education (ref = medium–high)													
Low					0.98	*	0.59				0.12		0.21
Mother’s education (ref = medium–high)													
Low					−1.64	***	0.61				−0.47	**	0.21
*Work 1 year after first birth*													
Constant					−3.99	***	0.64				−2.50	***	0.30
Calendar time (ref = 1998–2003)													
<1994					−0.56		0.37				−0.19		0.25
1994–1998					−0.38		0.34				0.14		0.18
2003–2006					–	–	–				−0.37	*	0.19
Educational attainment (ref = primary)													
Tertiary					2.50	***	0.68				2.07	***	0.28
Secondary					0.53	*	0.31				0.44	**	0.17
Still in education					1.47		0.92				0.01		0.31
Cumulated work experience at first conception (ref = never worked)													
(0–2) years					5.62	***	0.52				1.23	***	0.20
(2–4) years					7.10	***	0.58				1.79	***	0.21
(4–6) years					8.03	***	0.62				2.00	***	0.26
(6–8) years					8.81	***	0.69				2.41	***	0.35
>8 years					8.44	***	0.65				2.71	***	0.39
Number of non-employment spell at first conception (ref = 1)													
0.00					1.54	**	0.63				0.33	*	0.18
≥2					−3.22	***	0.39				−1.30	***	0.20
Father’s education (ref = medium–high)													
Low					0.27		0.64				0.45	**	0.21
Mother’s education (ref = medium–high)													
Low					−0.45		0.70				−0.20		0.20
*Employment entry after first child birthday*													
Age of the first child (spline), ref = 0													
Constant					−4.22	***	0.53				−2.92	***	0.33
1–2					0.71	*	0.42				0.75	***	0.27
2–3					0.12		0.34				0.59	***	0.19
3+					−0.01		0.13				−0.07		0.07
Calendar time (spline, ref = 2003)													
<1994					0.06		0.15				0.21		0.25
1994–1998					0.04		0.11				0.01		0.08
1998–2003					0.04		0.07				−0.04		0.05
2003–2006					–	–	–				−0.06		0.07
Educational attainment (ref = primary)													
Tertiary					3.64	***	0.59				1.56	***	0.23
Secondary					1.17	***	0.29				0.47	***	0.15
Still in education					−0.39		0.99				−0.39		0.28
Cumulated work experience at first conception (ref = never worked)													
(0–2) years					2.88	***	0.62				0.14		0.18
(2–4) years					3.47	***	0.73				0.48	**	0.19
(4–6) years					3.93	***	0.86				0.59	**	0.25
(6–8) years					5.19	***	0.89				0.49		0.37
>8 years					5.14	***	0.81				0.18		0.45
Number of non-employment spell at first conception (ref = 1)													
0.00					1.48	**	0.65				0.26		0.17
≥2					−0.39		0.55				−0.21		0.17
Father’s education (ref = medium–high)													
Low					0.35		0.49				0.01		0.19
Mother’s education (ref = medium–high)													
Low					−0.82		0.54				−0.22		0.18
Standard deviations of unobserved heterogeneity terms													
Fertility					0.95	***	0.16				0.64	***	0.15
Employment					1.78	***	0.21				0.84	***	0.12
Correlation between unobserved heterogeneity terms													
Fertility-Employment					0.24	**	0.12				0.52	***	0.20

* 10 %; ** 5 %; *** 1 %

## Footnotes

1 These regulations were in force until the end of 2007, i.e., in the time covered by our empirical analysis.

2 For the specification of these baseline hazard durations we used a piecewise linear spline (also known as a generalized Gompertz formulation), instead of the more common piecewice constant specification. A linear spline is a flexible form of representing the effect of a continuous independent variable. With sufficient bend points, this specification makes it possible to efficiently capture any log-hazard pattern in the data.

3 The results of the sensitivity tests were not presented here, but are available from the authors on request.
